# Dietary intervention rescues a bone porosity phenotype in a murine model of Neurofibromatosis Type 1 (NF1)

**DOI:** 10.1371/journal.pone.0304778

**Published:** 2024-06-24

**Authors:** Alexandra K. O’Donohue, Xiaoying C. Li, Lucinda R. Lee, Emily R. Vasiljevski, David G. Little, Craig F. Munns, Aaron Schindeler

**Affiliations:** 1 Bioengineering and Molecular Medicine Laboratory, The Children’s Hospital at Westmead and the Westmead Institute for Medical Research, Westmead, New South Wales, Australia; 2 The Children’s Hospital at Westmead Clinical School, Faculty of Medicine and Health, The University of Sydney, Sydney, New South Wales, Australia; 3 School of Chemical and Biomolecular Engineering, Faculty of Engineering, The University of Sydney, Sydney, New South Wales, Australia; 4 Child Health Research Centre, Faculty of Medicine, The University of Queensland, Brisbane, Queensland, Australia; 5 Department of Endocrinology and Diabetes, Queensland Children’s Hospital, Brisbane, Queensland, Australia; BSRC Alexander Fleming: Biomedical Sciences Research Center Alexander Fleming, GREECE

## Abstract

Neurofibromatosis type 1 (NF1) is a complex genetic disorder that affects a range of tissues including muscle and bone. Recent preclinical and clinical studies have shown that *Nf1* deficiency in muscle causes metabolic changes resulting in intramyocellular lipid accumulation and muscle weakness. These can be subsequently rescued by dietary interventions aimed at modulating lipid availability and metabolism. It was speculated that the modified diet may rescue defects in cortical bone as NF1 deficiency has been reported to affect genes involved with lipid metabolism. Bone specimens were analyzed from wild type control mice as well as *Nf1*_*Prx1*_^-/-^ (limb-targeted *Nf1* knockout mice) fed standard chow versus a range of modified chows hypothesized to influence lipid metabolism. Mice were fed from 4 weeks to 12 weeks of age. MicroCT analysis was performed on the cortical bone to examine standard parameters (bone volume, tissue mineral density, cortical thickness) and specific porosity measures (closed pores corresponding to osteocyte lacunae, and larger open pores). *Nf1*_*Prx1*_^-/-^ bones were found to have inferior bone properties to wild type bones, with a 4-fold increase in the porosity attributed to open pores. These measures were rescued by dietary interventions including a L-carnitine + medium-chain fatty acid supplemented chow previously shown to improve muscle histology function. Histological staining visualized these changes in bone porosity. These data support the concept that lipid metabolism may have a mechanistic impact on bone porosity and quality in NF1.

## Introduction

Neurofibromatosis Type 1 (NF1) is a genetic condition that features nerve-associated tumors but can also include a range of characteristic non-tumor manifestations [1]. The *NF1* gene encodes for the protein neurofibromin, a tumor suppressor that negatively regulates the p21Ras GTPase. Functional loss of neurofibromin results increased p21Ras signaling [2]. In the context of tumors, this leads to dysregulated cell growth but the role of p21Ras in musculoskeletal tissues (including bone) can be more ambiguous [3]. Still, the musculoskeletal complications of NF1 can be profound and include osteopenia and osteoporosis [4], focal orthopedic complications [5], and impacts on muscle strength and fatiguability [6]. The biological mechanisms underlying the pathobiology of NF1 in bone and muscle are largely informed by mouse models, particularly the limb-specific *Nf1*_*Prx1*_^-/-^ knockout mice [7].

*Nf1* null cortical bone from *Nf1*_*Prx1*_^-/-^ mice exhibits a reduced mass and increased porosity. Bone porosity can be described in terms of macropores (which are 100 μm or higher and can be readily seen on radiographic images) and micropores (such as normal osteocyte lacuna that are typically 7–30 μm). NF1 porosity phenotype features both large macropores as well as an increased microporosity from enlarged osteocyte lacunae [8]. Subsequent analysis of bone from *Nf1*_*Prx1*_^-/-^ and *Nf1*_*Col1*_^-/-^ mouse models suggested not only a hindering of osteocyte development but also a problematic persistence of blood vessels within the cortical bone. This was later linked to the upregulation of *Mgp (matrix gla protein)* that was speculated to underpin the hyperosteoidosis associated with osteoblasts and endothelial vessel cells [9].

While the muscle-specific *Nf1*_*MyoD*_^-/-^ knockout mouse was the first model to reveal an accumulation of intramyocellular lipid [10], a comparable phenotype was reported in *Nf1*_*Prx1*_^-/-^ mouse muscle [11]. The *Nf1*_*Prx1*_^-/-^ muscle phenotype could be rescued using a dietary intervention enriched for medium-chain fatty acids (MCFAs) and L-carnitine, showing a dramatic decrease in muscle lipid (71%) and a partial improvement in grip strength [12]. A subsequent 2020 study trialed several variations of this dietary intervention [13]. This included L-carnitine and MCFAs separately and in combination, an intermittent dosing regimen (5/7 days), a mitochondrial cocktail (L-carnitine, CoQ10, creatine and riboflavin), and a low-fat diet. A 2021 Phase 2A clinical trial revealed improved 6-minute walk test and standing jump distance in a cohort of six children (ages 9–12) placed on 1000 mg/day carnitine to treat their muscle weakness and fatigue [14].

To date, there has been no definitive links between the metabolic changes associated with NF1 muscle weakness and the fundamental bone mineralization issues associated with NF1 deficiency. While the NF1 bone phenotype has been correlated with deficient osteogenesis [8], microarray analysis examining cortical bone samples from *Nf1*_*Prx1*_^-/-^ and *Nf1*_*Col1*_^-/-^ did show a disruption of lipid metabolism genes [9]. To investigate the potential role of diet and dietary rescue on bone, spine and long bone specimens were analyzed from our prior study comparing the impact of different dietary interventions on *Nf1*_*Prx1*_^-/-^ mouse muscle [13].

## Materials and methods

### Animal ethics and husbandry

*Prx-Cre* transgenic mice [15] supplied by Jackson Laboratory (Bar Harbor, ME, USA) and *Nf1*^*flox/flox*^ mice [16] supplied by the National Cancer Institute (Bethesda, MD, USA) were crossbred to generate *Nf1*_*Prx1*_^-/-^ mice after two generations of breeding. Mice were bred at the Children’s Hospital at Westmead Transgenic Facility and were given access to standard rodent chow and water ad libitum. Genotyping was performed by quantitative real-time PCR for the *Cre* and *Nf1*^*flox*^ alleles by TransnetYX Inc (Cordova, TN, USA). *Nf1*_*Prx1*_^-/-^ mice are smaller than their littermates and were given daily 0.1 mL saline injections until four weeks of age, which was found to improve their health. All animal experiments were approved by the local Animal Ethics Committee under protocol K319.

### Study design and dietary formulae

**Fig 1:**
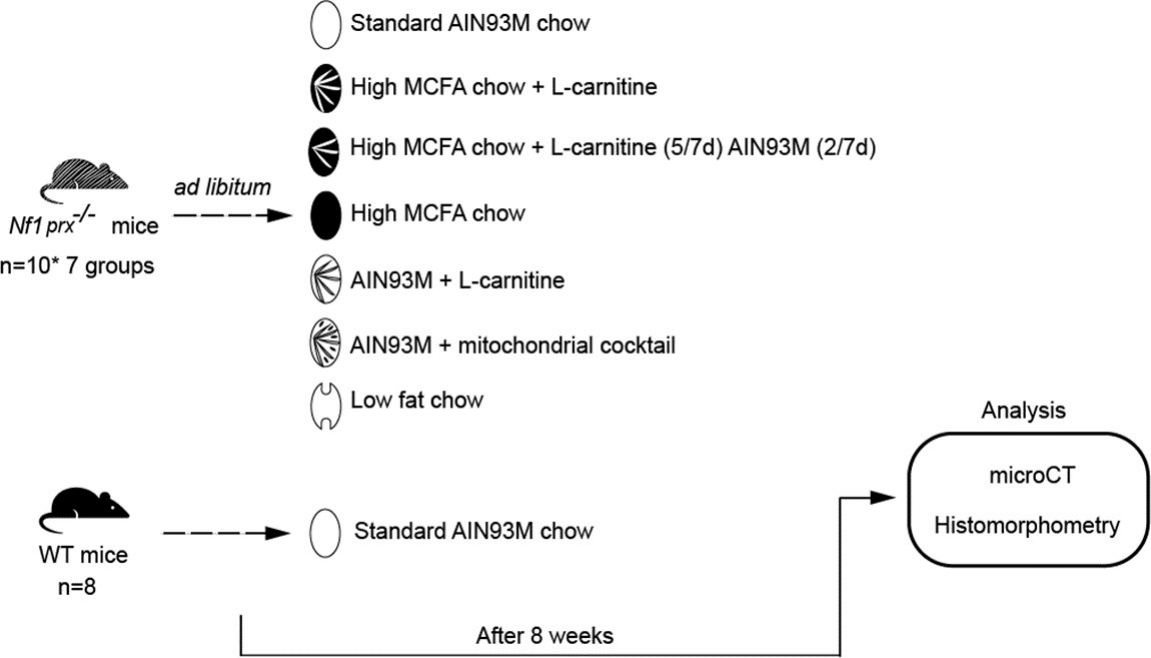
The overview of the dietary intervention and outcome assessments involved in the NF1 study. A total of 70 *Nf1*_*Prx1*_^-/-^ mice were fed with a standard AIN93M chow or one six modified chows for 8 weeks. 8 wild type mice fed AIN93M chow were included as controls. Tibial samples were harvested for microCT analysis and histomorphometry.

Bone specimens were analyzed from a prior published mouse study that examined the *Nf1*_*Prx1*_^-/-^ muscle phenotype in response to dietary interventions [13]. To summarize, n = 70 female *Nf1*_*Prx1*_^-/-^ mice (n = 10/group) were fed *ad libitum* either standard AIN93M rodent chow, or one of six other AIN93M-modified diets altered to reduce LCFA intake and improve lipid metabolism (Fig 1). Feeding started at 4 weeks. Cages of *Nf1*_*Prx1*_^-/-^ mice were randomly assigned to the dietary treatment groups. Female WT littermates (n = 8) were fed standard AIN93M chow. After eight weeks of dietary intervention, mice were euthanized via cervical dislocation. Tibiae were wrapped in saline soaked gauze and stored at -80°C for radiographic and histomorphometry analysis, with micro-computed tomography analysis as the primary outcome.

Details of the ingredients of the therapeutic diets have been stated in the original muscle paper [13]. Briefly, standard AIN93M rodent chow pellets contained digestible energy, carbohydrates, protein, and total fats. All modified diet formulas were designed to have a base nutritional composition equivalent to AIN93M, except for the low-fat chow. The low-fat diet had 1.8% fat, which was compensated for by a higher amount of carbohydrates (68.6%) and 15.0 MJ/kg of digestible energy. Addition of supplements was based on standard chow consumption rates (Specialty Feeds, WA, Australia). L-carnitine was added to AIN93M or high MCFA chow at a concentration of 1.71 g/kg to achieve an optimal daily dose of 300 mg/kg for rodents. The L-carnitine/MCFA diet was also given 5/7 days per week to model the effect of regular ‘cheat days’. The mitochondrial cocktail chow was supplemented by L-carnitine, CoQ10 (20 mg/kg/mouse/day), creatine (10 mg/kg/mouse/day), and riboflavin (active vitamin B2; 12 mg/kg/mouse/day). Octanoic acid (C8:0, 2.8%) was included as the predominant lipid source in the high MCFA chow. Chows derived from AIN93M and the low-fat chow utilized LCFAs (≥C16:0) as their fat source.

### Micro-computed tomography (microCT) analysis

Bone samples were analyzed *ex vivo* with high (3.2 μm voxel) resolution by microCT (Bruker SkyScan 1272, Kontich, Belgium). Right tibiae (n = 78) were imaged at an isotropic voxel resolution of 3.2 μm, exposure time of 3500 ms, using 0.5 mm aluminium filter, 50 kV x-ray tube voltage, and 200 μA tube electric current. All cross-sectional images were reconstructed using a 0–0.11 greyscale (NRecon Software version 1.1.17; Bruker) and straightened by DataViewer Software version 1.5.6.2 (Bruker). Analysis of bone morphometric parameters was performed using CTAn Software version 1.17.7.2 (Bruker).

The volume of interest (VOIs) for analyzing the cortical bone tissues were defined in CTAn according to mouse genotype. For WT group cortical bone VOI commenced at 5 mm below the metaphyseal growth plate and distally extended for another 0.5 mm. For *Nf1*_*Prx1*_^-/-^ tibiae (all treatment groups), the cortical bone VOI was defined to start from 3mm below the growth plate due to the smaller bone size. The segmentation threshold for 3D morphometric analysis was determined using a density-based, global threshold of 0.4 grams hydroxyapatite (gHA)/cm^3^. Major bone morphometric parameters calculated by CTAn and reported in bone analysis included: bone volume (BV), bone volume fraction (BV/TV), tissue mineral density (TMD), and cortical thickness (Ct.Th). Representative 3D images of the tibia coronal cut were obtained from Drishti Volume Exploration and Presentation Software version 1.0. In addition, parameters associated with closed and open intracortical pores were analyzed for NF1 based on protocols supplied by Bruker microCT Systems (Dr Phil L Salmon) MN058 “Osteocyte and blood vessel analysis in cortical bone”, MN010 “3D visualization of open and closed porosity”, MN011 “Advanced Porosity Analysis”, MN059 “Porosity analysis”. The workflow involved making a white mask of the cortical bone on slices within the ROI (cortical bone with porosity), performing a closing operation and coarse despeckling (cortical bone, no porosity), and comparing with a fine despeckling function of <300 voxels (cortical bone, no small pores). Generated 3D models can be opened and overlayed in CTVol with the blood vessels colored red and osteocytes blue.

### Tibial length measurement

For *Nf1*_*Prx1*_^-/-^ mice and their WT littermates, the measurement of right tibial length was taken from X-ray images (Faxitron MX-20, Wheeling IL, USA) corresponding to the distance from the middle of the proximal articular surface to the distal-most projection of the medial malleolus. The average of three independent measurements was taken for each specimen.

### Bone histological analysis

From results of the tibial open pore porosity microCT analysis, the median specimens were chosen and fixed in 10% neutral buffered formalin (NBF) overnight and subsequently decalcified in *in situ* decal solution (0.34 M EDTA, PFA, pH 8.0) for two days and then 0.34 M EDTA (pH 8.0) for 10 days at 4°C. Decalcified femurs were then paraffin-embedded and cut to 5 μm sections.

Histological staining was performed on the sections following standard protocols. Ploton silver nitrate stain was performed to stain the lacuna-canalicular network as previously described by Mazur *et al*. [17]. Matrix Gla protein (MGP) immunohistochemistry, a local calcification inhibitor secreted primarily by chondrocytes and vascular smooth muscle cells [18], was also performed to identify any correlations between MGP expression and pore size. Specimens were treated as previously described [19], substituting antibodies with an anti-mouse MGP primary (1:100 dilution; 1 mg/mL #PA5-76797 Thermo Fisher Scientific, MA, USA) and a biotinylated Goat Anti-Rabbit (1:200 dilution; BA-1000-1.5, Vector Laboratories, CA, USA). Slides were counterstained with Harris hematoxylin and imaged using the Aperio CS2 slide scanner (Leica, Germany).

### Statistical analysis

Data are expressed as the mean ± SD, with medial representative images used for representative histology. Four bones were excluded from analysis (WT N = 1, *Nf1*_*Prx1*_^-/-^ N = 3) due to damage to the growth plate during harvesting that prevented definition of the VOIs. One-way analysis of variance (ANOVA) testing was carried out to calculate the statistical significance of obtained data. Dunnett’s post hoc multiple comparisons tests were performed on the datasets to identify differences between each group and the *Nf1*_*Prx1*_^-/-^ standard control group. All tests were performed on GraphPad Prism 9 (GraphPad Software, San Diego, USA) with a p<0.05 considered statistically significant. Blinding of genotype and/or treatment was infeasible during treatment but was applied where possible during post-study analysis.

## Results

### Dietary interventions affect cortical bone measures in NF1 mice

**Fig 2:**
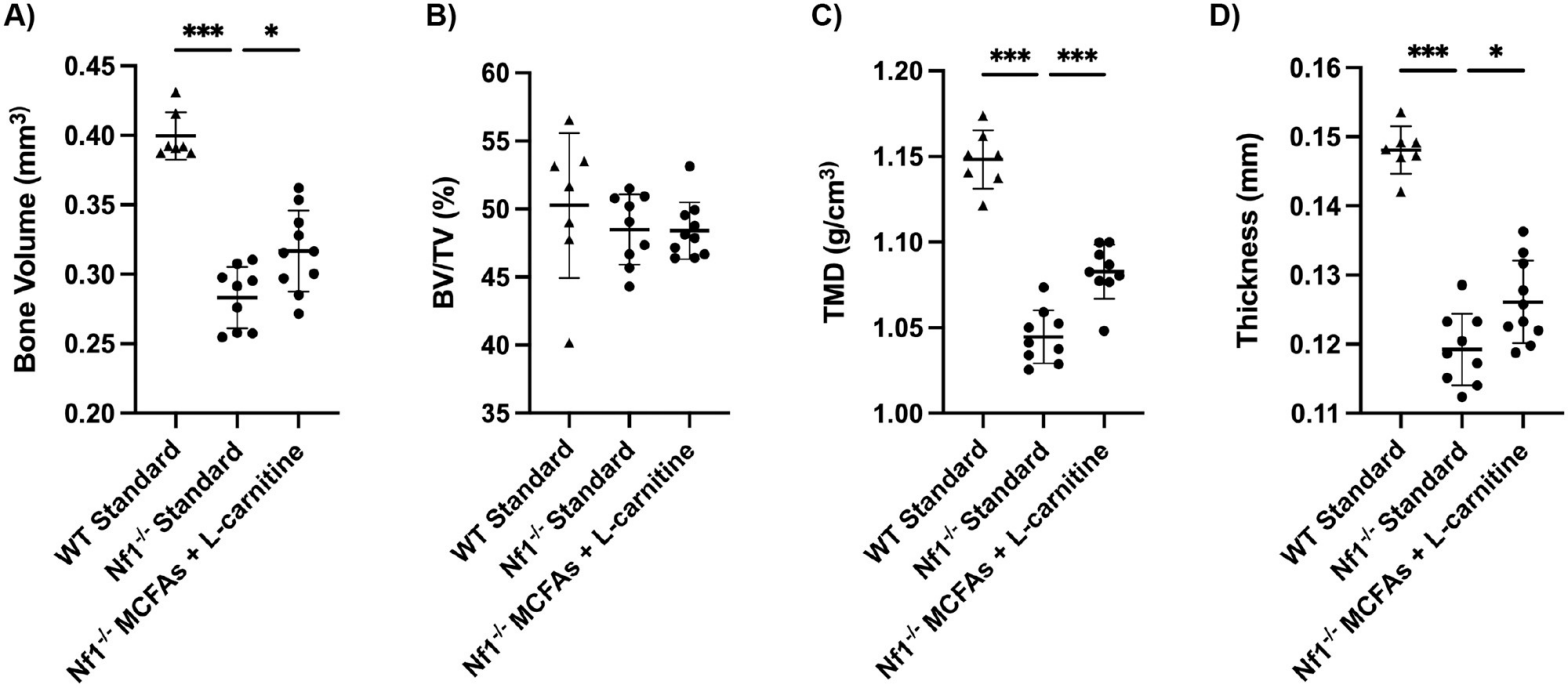
MicroCT assessment of the mid-diaphyseal cortical bone of tibiae from the WT controls and *Nf1Prx1-/-* mice fed with MCFAs and L-carnitine. Outcome measures include (A) bone volume (BV), (B) bone volume/tissue volume (BV/TV), (C) tissue mineral density (TMD), (D) cortical thickness. Data were analyzed using a one-way ANOVA with multiple comparisons Dunn’s test comparing all experimental groups to *Nf1Prx1-/-* standard control group, * = p<0.05, *** = p<0.001.

MicroCT analysis of tibial specimens encompassed cortical bone volume (BV), bone volume fraction (BV/TV), cortical tissue mineral density (TMD) and cortical thickness (Ct.Th). All of these metrics (BV, TMD, Ct.Th) were significantly decreased in *Nf1*_*Prx1*_^-/-^ bones compared to wild type bones when both groups were fed an equivalent standard diet (AIN93M chow) (Fig 2). The smaller bone size was also seen by radiographic measurement of tibial length (S1 Fig).

The primary treatment group was the standard MCFA/L-carnitine diet, which had previously been shown to impact on muscle lipid and function [12]. Significant improvements were seen in cortical TMD and cortical thickness with the dietary intervention (p<0.001, p<0.05 respectively) (Fig 2). Analysis of other groups showed significant increases in cortical BV with MCFA/L-carnitine diet (5/7), MCFAs alone, L-carnitine alone, and with the Mito mix, cortical thickness with the MCFA/L-carnitine diet, MCFA/L-carnitine (5/7), and L-carnitine alone, and cortical TMD was significantly increased with the MCFA/L-carnitine diet, MCFAs alone and with the low-fat diet (S2 Fig).

### Dietary interventions significantly reduce cortical porosity in NF1 mice

**Fig 3:**
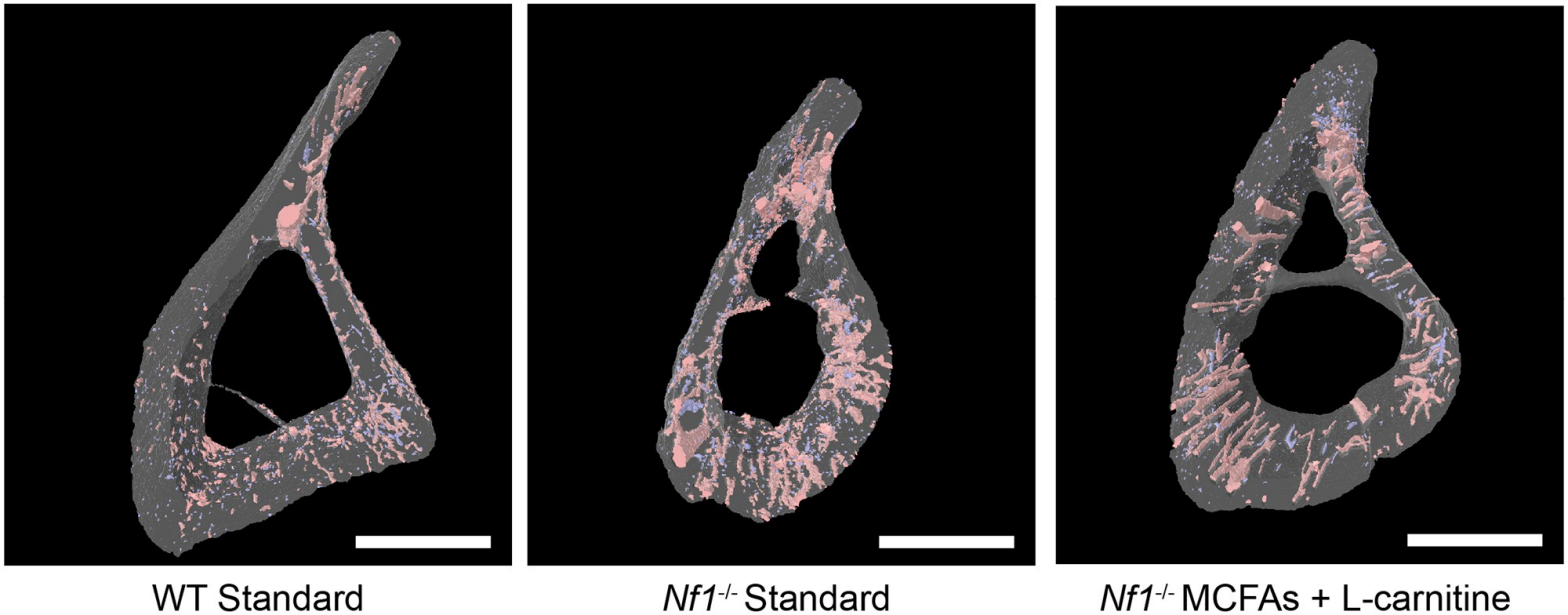
Three-dimensional reconstructions of the mid-diaphyseal cortical bone with open (red) and closed pores (blue) detected by microCT scans. Representative images demonstrate cortices with a median total pore porosity from each group. Open/vascular pores are illustrated in red. Closed/osteocyte lacunar pores are in blue.

Prior reports on the *Nf1*_*Prx1*_^-/-^ bone phenotype highlighted major changes in bone porosity, and this was associated with larger lacunae, increased blood vessel invasion, and under-mineralization [8]. To visualize the effects of *Nf1* deficiency on bone, 3-dimensional reconstructions were created for tibial bone specimens using microCT analysis to reconstruct bone pores (Fig 3, S3 Fig for exemplars from all treatment groups). These pores were segregated into open pores (corresponding to blood vessels) and closed pores (osteocyte lacunae) based on size. Comparison of the wild type and *Nf1*_*Prx1*_^-/-^ specimens fed AIN93M chow indicated the smaller size of the NF1 bones as well as the increased presence of open pores in the cortical bone.

**Fig 4:**
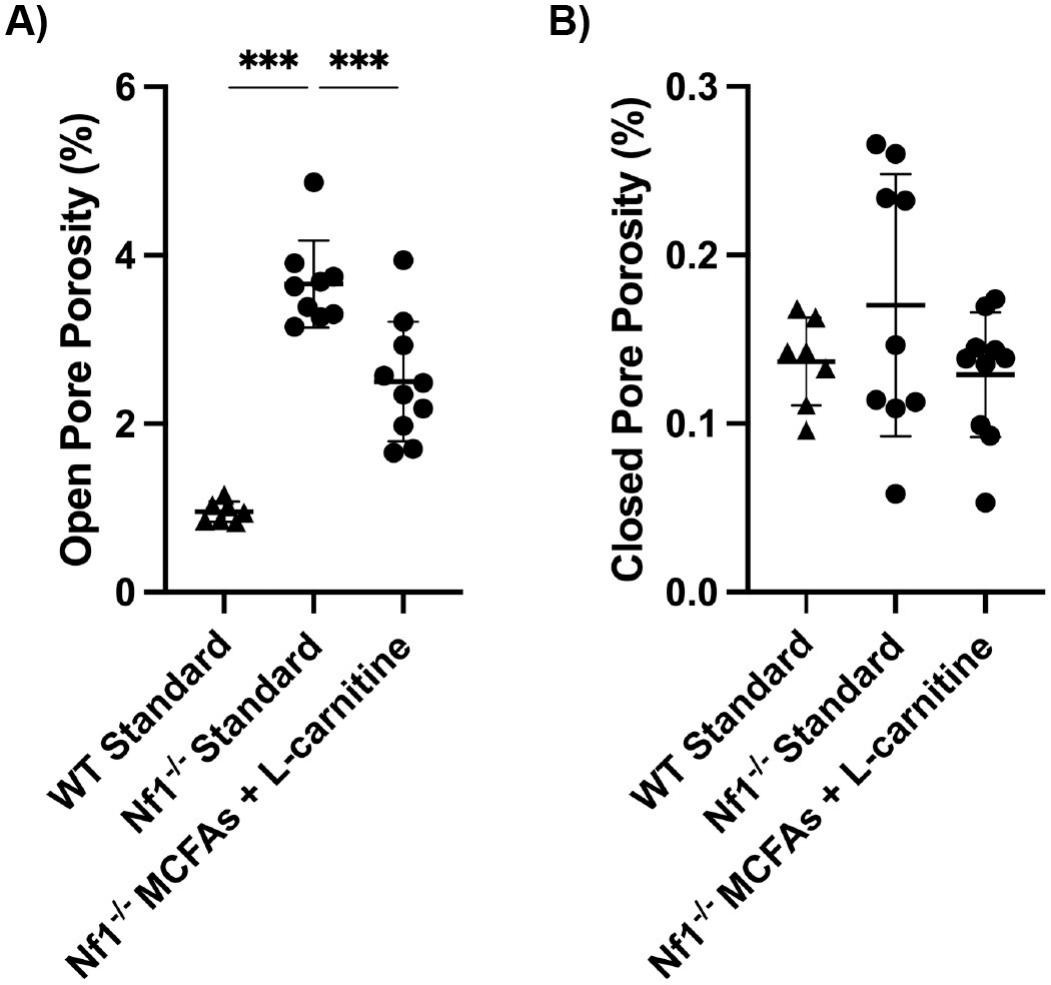
MicroCT porosity analysis of the mid-diaphyseal cortical bone of tibiae from the WT controls and *Nf1Prx1-/-* mice fed with different therapeutic diets. Outcome measures include (A) % open pore porosity (normalized by total volume), and (B) % closed pore porosity (normalized by total volume). Data were analyzed using a one-way ANOVA with multiple comparison corrected by statistical hypothesis testing, *** = p<0.001.

Pore volumes were quantified for all specimens and subjected to statistical analysis. To avoid any confounding effects of bone size on pore calculations, porosity was normalized to bone total volume (TV) to give a percentage (%) value. For open pores, the *Nf1*_*Prx1*_^-/-^ bones showed ~4-fold greater porosity than wild type bones (Fig 4). This was significantly decreased for other dietary interventions (32% reduction in open pore porosity with L-carnitine and MCFAs, with reductions in other diets ranging from 27–44%). The closed pores (corresponding to lacunae) did not show a significant difference between wild type and *Nf1*_*Prx1*_^-/-^ samples and were unaffected by diet. Notably, the porosity volume taken up by closed pores was at least one order of magnitude less than that of the open pores. The full dataset for all dietary interventions is available in S4 Fig.

### Qualitative histology visualizing the impact of L-carnitine on the bone matrix

**Fig 5:**
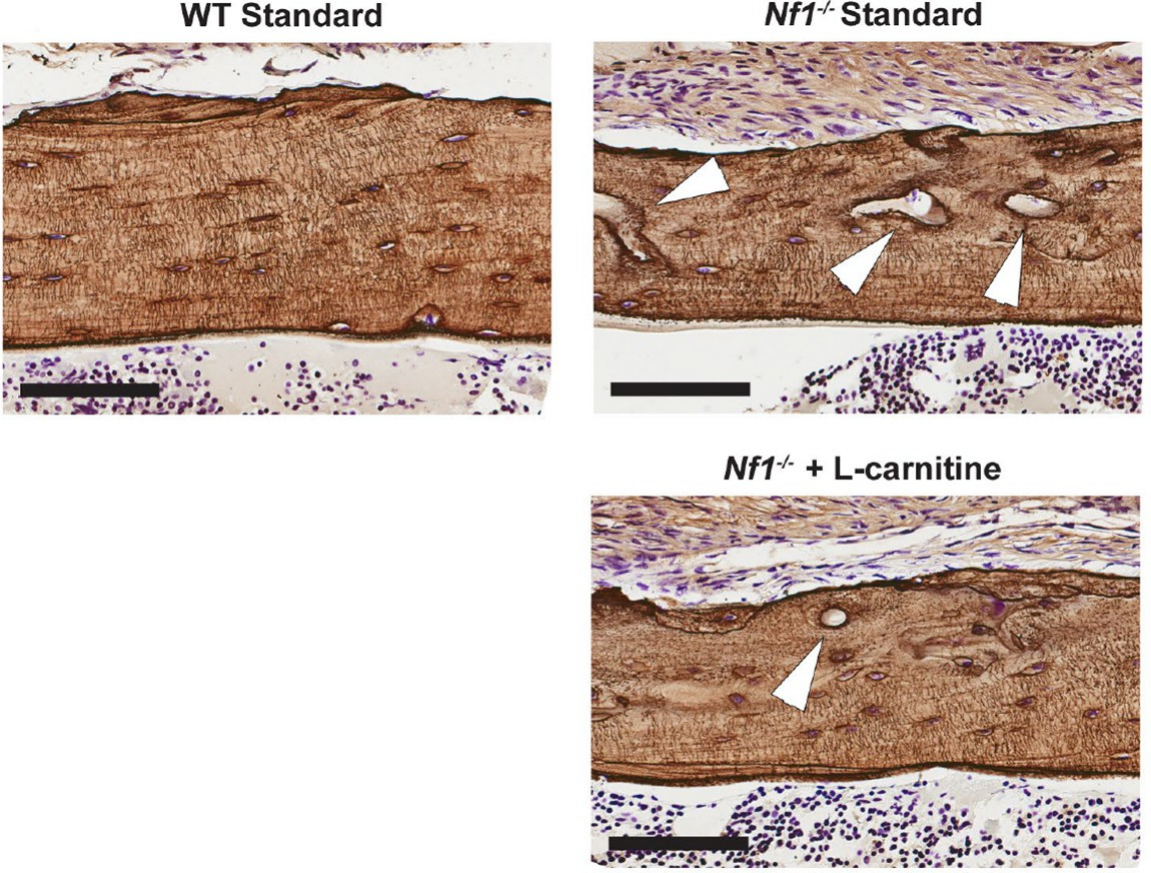
Bone histomorphology demonstrating pore porosity of WT controls and *Nf1Prx1-/-* mice fed with normal diet and the L-carnitine dietary intervention. Ploton silver staining on paraffin-sections of cortical bones highlights the large pores (white arrows) are most prominent in the untreated *Nf1Prx1-/-* mice. The thin lines connecting the osteocytes illustrate staining of the canalicular network. Cells were counterstained with 0.1% cresyl violet.

Histological staining was used to visualize the macro-vascular pores and osteocyte lacunar pores within the cortical bone matrix. The Ploton silver staining histology demarked the mineralization front and revealed the invading blood vessels, osteocyte lacunae, and osteocyte canaliculi (Fig 5). The *Nf1*_*Prx1*_^-/-^ bone lacunae appeared slightly larger and less flattened than the wild type bone lacunae, consistent with prior reports [8, 9].

MGP is a negative regulator of calcification [18] that is reportedly upregulated in NF1 deficient bone [9]. We hypothesized that MGP may be upregulated in the local vicinity of pores, particularly large pores associated with blood vessel invasion. Immunohistochemical staining for MGP revealed staining adjacent to some large pores in *Nf1*_*Prx1*_^-/-^ samples, however the staining was highly variable both between samples and even within the same specimen (S5 Fig). Moreover, MGP was seen not only adjacent to vessels but also in regions of the bone matrix and growth plate cartilage. While dietary intervention appeared to change the frequency of large pores, MGP staining was not noticeably affected.

## Discussion

The role of NF1 in muscle and bone is complex, as NF1-Ras signaling can affect progenitor cell recruitment, proliferation, and differentiation, as well as the function of mature tissues. Initial studies focused on the reduced osteogenic capacity of osteoprogenitors [20] and the increased resorptive potential of NF1 deficient osteoclasts [21]. However, subsequent studies have used a variety of tissue-targeted double-knockout mice to further study the mechanism of NF1 deficient bone. These include osteoprogenitor-targeted *Nf1*_*Osx*_^-/-^ mice [22], osteoblast-targeted *Nf1*_*Col1*_^-/-^ mice [9], and osteocyte-targeted *Nf1*_*Dmp1*_^-/-^ mice [23]. A common critique of these studies is to question the relevance of double-knockout models. While focal bone defects (e.g. a tibial pseudarthrosis) are associated with double inactivation of NF1 [24], it is likely that the systemic osteopenia seen in some NF1 individuals is due to haploinsufficiency. Still, a common feature of NF1 deficient mouse models is that double inactivation can be beneficial in revealing underlying mechanisms. For example, the intramyocellular lipid accumulation seen in *Nf1*_*MyoD*_^-/-^ mice [9] was later found to be present in NF1 patient biopsies [25]. Still, care must be taken in interpreting the data in the context of human disease. Another issue is the question of using only female mice, though this is common when including both sexes would increase variation and decrease statistical power. Notably, a 2022 study utilizing an *Osx* promoter driven conditional NF1 knockout mouse found negative changes in bone were independent of sex [26]. Moreover, selecting a single mouse sex allowed us to power the study appropriately while allowing for group housing.

This study utilized bone specimens harvested from a prior study [13] focused on rescuing the NF1 muscle phenotype. Our approach highlights the utility of collecting all potential tissues of interest to allow for subsequent analysis. The prior dietary rescue studies suggest an underlying mechanism–that NF1 deficiency can affect the translocation of long chain fatty acids across the mitochondrial membrane. This process is generally facilitated by L-carnitine. Primary Carnitine Deficiency (PCD) shows a similar pattern of skeletal muscle weakness and lipid accumulation and is also treatable with dietary carnitine supplementation [25]. The concept that this could also impact on bone is novel and unexpected–compared to bone, muscle is a highly metabolically active tissue with high energy use.

For bone studies, there is value in performing biomechanical tests to translate structural (microCT) findings to functional outcomes such as bone strength. Standard 3-point tibial bending tests were precluded in this instance due to the significantly reduced size of NF1 bones, which impacts the practicality and reliability of testing and may confound any influence of porosity. Instead, future studies could examine micro-indentation, which may be impacted by increased bone porosity via effects on bone stiffness [27].

In conclusion, muscle weakness and fatigue are increasingly being appreciated as a feature of NF1 that can impact on quality of life, particularly in children [6, 28]. The concept of L-carnitine as a safe and low-cost intervention for NF1 muscle weakness [14] may have profound effects moving forward, particularly as self-medication is straightforward. The findings of this study raise the possibility that L-carnitine may have secondary beneficial effects on bone. While a significant proportion (~30%) of children with NF1 may have osteopenia [4], there is insufficient data to suggest that this leads to an increased fracture risk or should be tested for or treated. These data from the *Nf1*_*Prx1*_^-/-^ mice suggest that any future trials for L-carnitine treatment of NF1 muscle could also include a DEXA arm to assess any benefits on bone health.

## Supporting information

S1 Fig Tibia length as determined from x-ray images, with each specimen (data point) representing the mean of triplicate measurements. Data were analysed using a one-way ANOVA with a multiple comparisons Dunn’s test comparing all groups to the Nf1Prx1-/- standard control group, *** = p<0.001 vs all other groups.
(TIF)

S2 Fig MicroCT assessment of the mid-diaphyseal cortical bone of tibiae from the WT controls and Nf1Prx1-/- mice fed with different therapeutic diets. Outcome measures include (A) bone volume (BV), (B) bone volume / tissue volume (BV/TV), (C) tissue mineral density (TMD), (D) cortical thickness. Data were analysed using a one-way ANOVA with multiple comparisons Dunn’s test comparing all groups to Nf1Prx1-/- standard control group, * = p<0.05, ** = p<0.01, *** = p<0.001.
(TIF)

S3 Fig Three-dimensional reconstructions of the mid-diaphyseal cortical bone with open (red) and closed pores (blue) detected by microCT scans. Representative images demonstrate cortices with a median total pore porosity from each group. Open/vascular pores are illustrated in red. Closed/osteocyte lacunar pores are in blue. Scale bar represents 500 μm.
(TIF)

S4 Fig Correlation of MicroCT porosity analysis of the mid-diaphyseal cortical bone of tibiae and previously quantified lipid accumulation within the quadriceps of Nf1Prx1-/- mice fed either standard chow or MCFAs + L-carnitine modified diet. Lipid area was calculated by quantifying total Oil Red O staining as a percentage of total section area. Data were analysed using simple linear regression, with a R2 value of 0.09158.
(TIF)

S5 Fig MicroCT porosity analysis of the mid-diaphyseal cortical bone of tibiae from the WT controls and Nf1Prx1-/- mice fed with different therapeutic diets. Outcome measures include (A) % open pore porosity (normalised by total volume), and (B) % closed pore porosity (normalized by total volume). Data were analysed using a one-way ANOVA with multiple comparison corrected by statistical hypothesis testing, * = p<0.05, ** = p<0.01, *** = p<0.001.
(TIF)

S6 Fig Matrix Gla protein (MGP) immunohistochemical staining within the cortical bone of WT controls, Nf1Prx1-/- fed AIN93M chow, and Nf1Prx1-/- fed L-carnitine. Descriptive histology illustrates the high variability of MGP staining both in the bone matrix and associated with large pores in the cortical bone. Scale bar represents 50 μm.
(TIF)

S1 File Excel file of MicroCT plotted in Figs 2A-2D and 4A, 4B. (XLSX)
