# Supplementary figures and images for: Dietary intervention rescues a bone porosity phenotype in a murine model of Neurofibromatosis Type 1 (NF1)
Source: PLoS One. 2024 Jun 24;19(6):e0304778. doi: 10.1371/journal.pone.0304778 (PMC11195983; doi:10.1371/journal.pone.0304778)

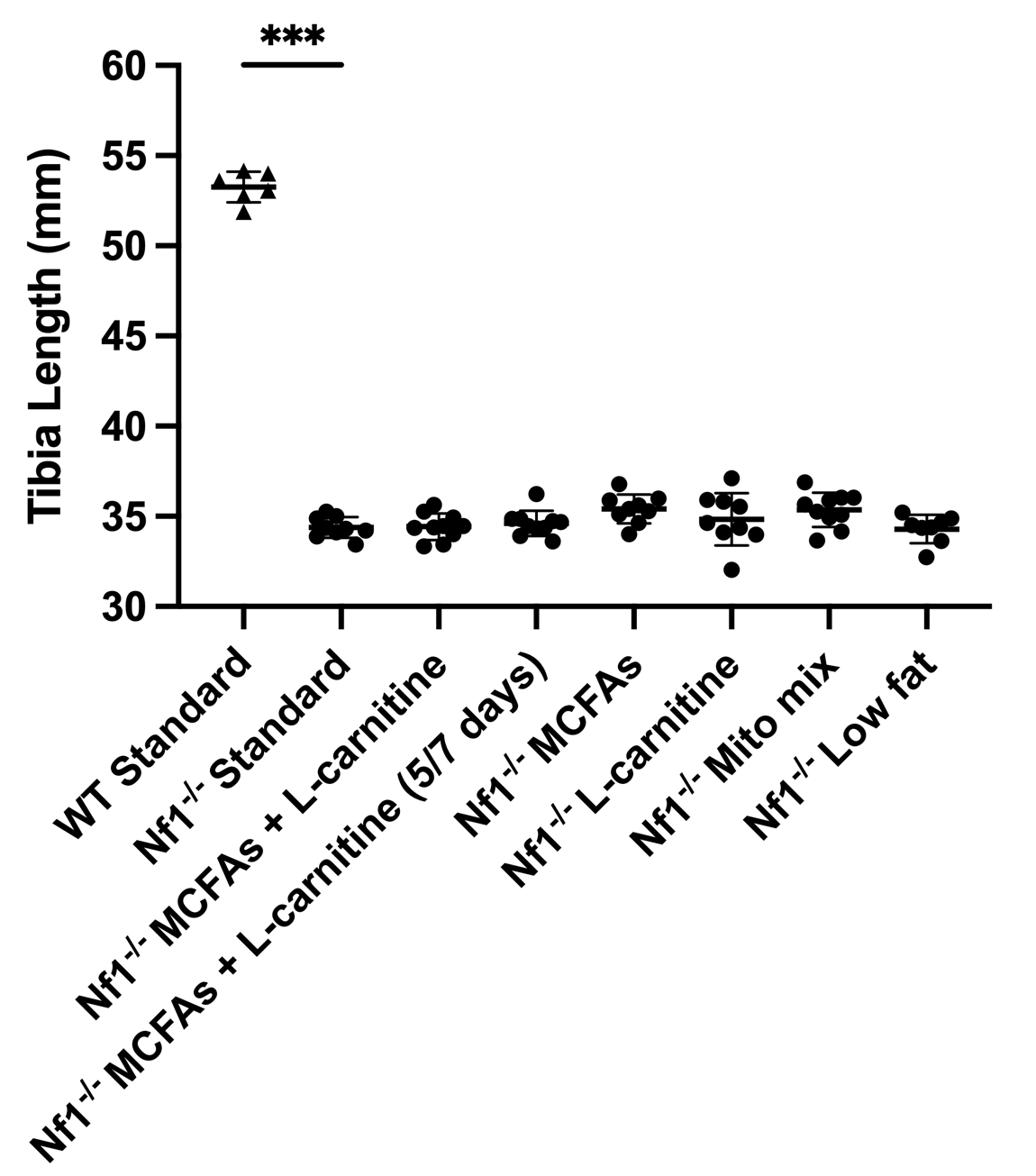

Supplement: S1 Fig — Data were analysed using a one-way ANOVA with a multiple comparisons Dunn’s test comparing all groups to the Nf1Prx1-/- standard control group, *** = p<0.001 vs all other groups. (TIF) [file pone.0304778.s001.tif]

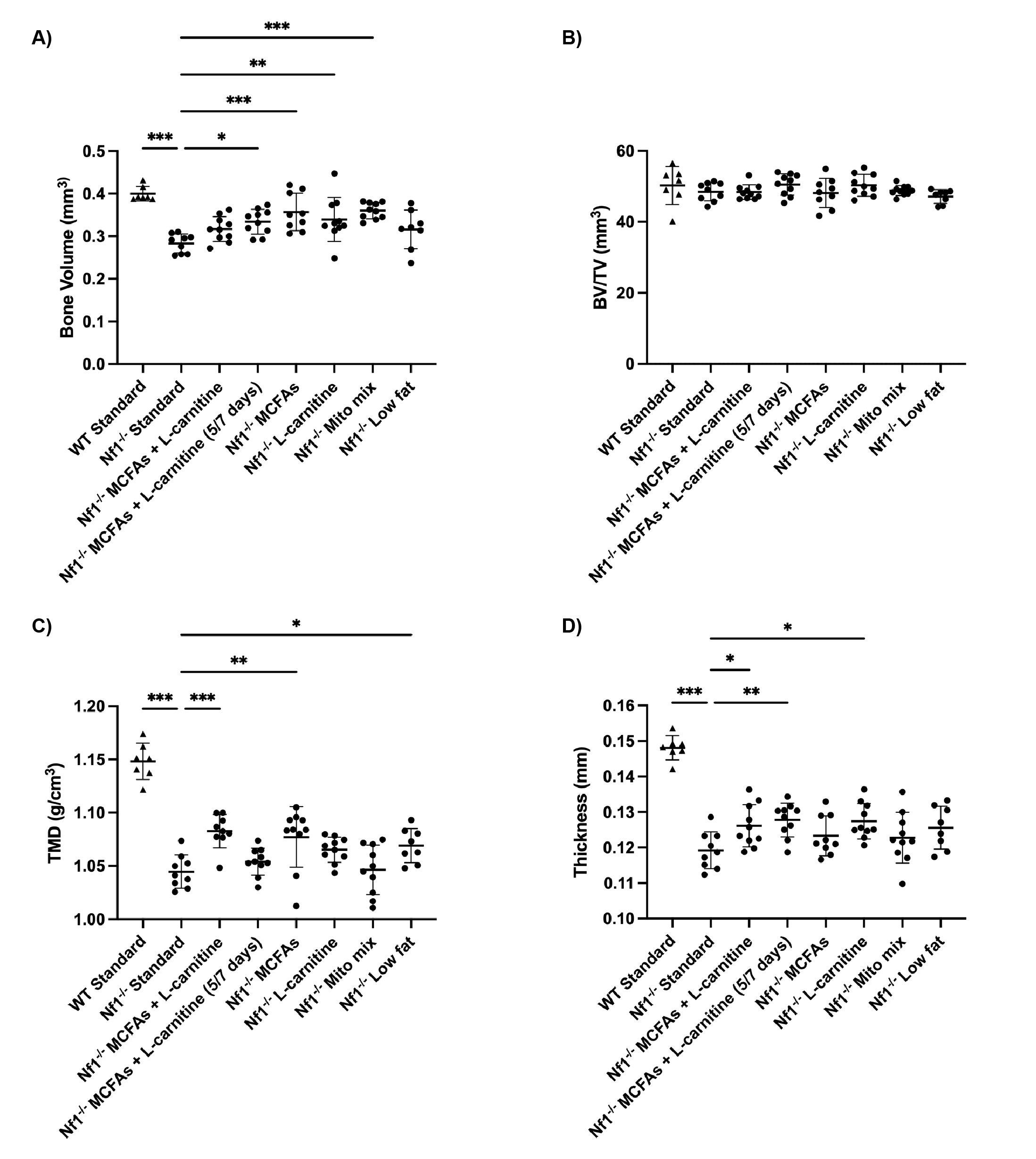

Supplement: S2 Fig — Outcome measures include (A) bone volume (BV), (B) bone volume / tissue volume (BV/TV), (C) tissue mineral density (TMD), (D) cortical thickness. Data were analysed using a one-way ANOVA with multiple comparisons Dunn’s test comparing all groups to Nf1Prx1-/- standard control group, * = p<0.05, ** = p<0.01, *** = p<0.001. (TIF) [file pone.0304778.s002.tif]

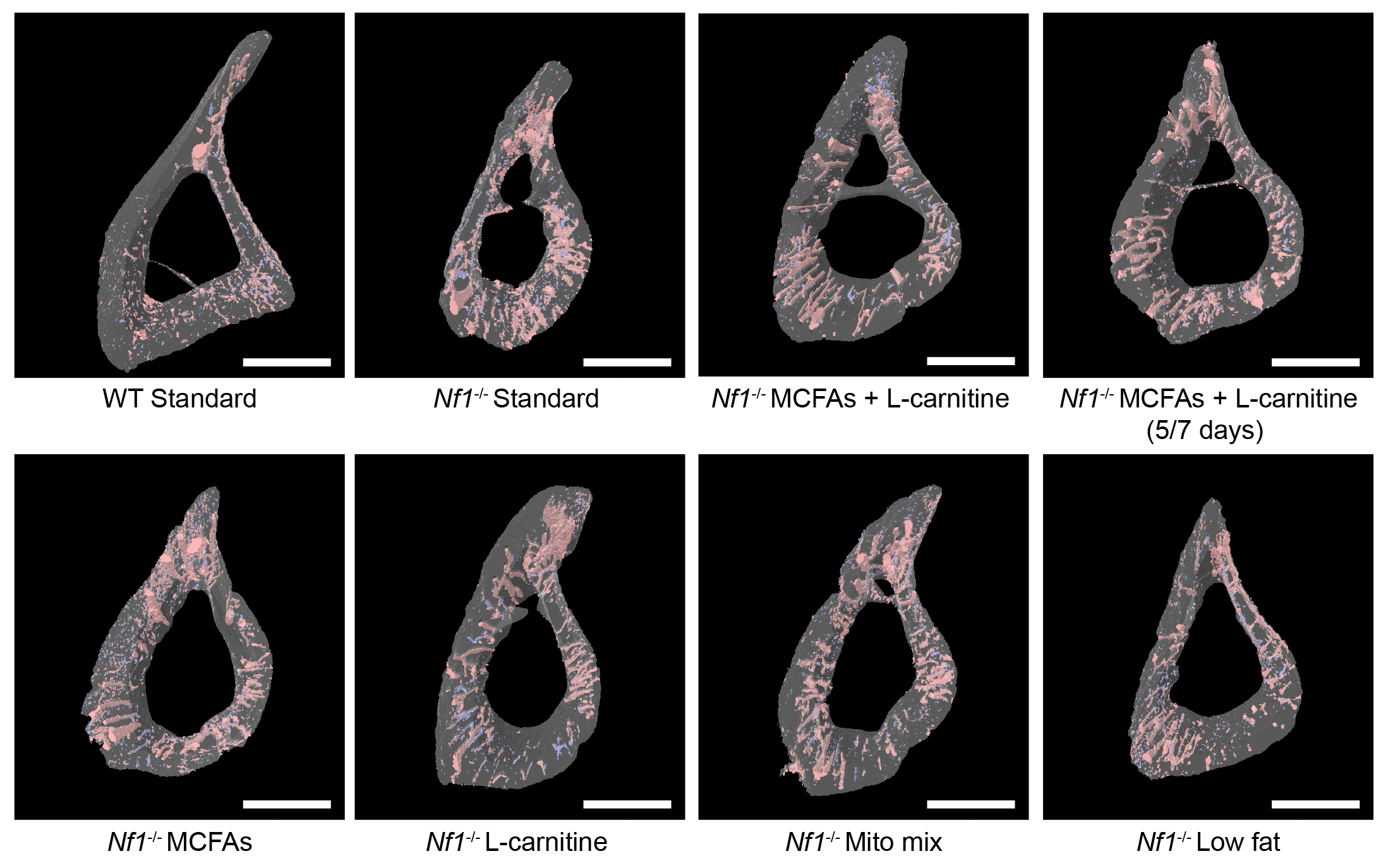

Supplement: S3 Fig — Representative images demonstrate cortices with a median total pore porosity from each group. Open/vascular pores are illustrated in red. Closed/osteocyte lacunar pores are in blue. Scale bar represents 500 μm. (TIF) [file pone.0304778.s003.tif]

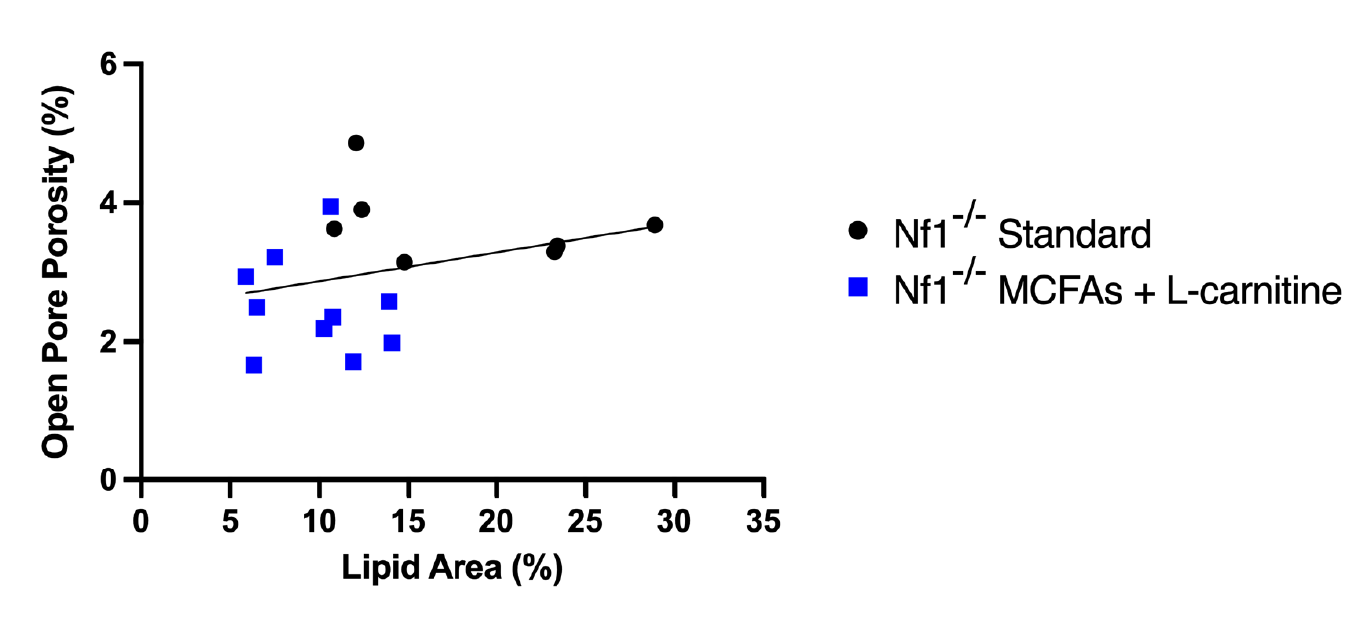

Supplement: S4 Fig — Lipid area was calculated by quantifying total Oil Red O staining as a percentage of total section area. Data were analysed using simple linear regression, with a R2 value of 0.09158. (TIF) [file pone.0304778.s004.tif]

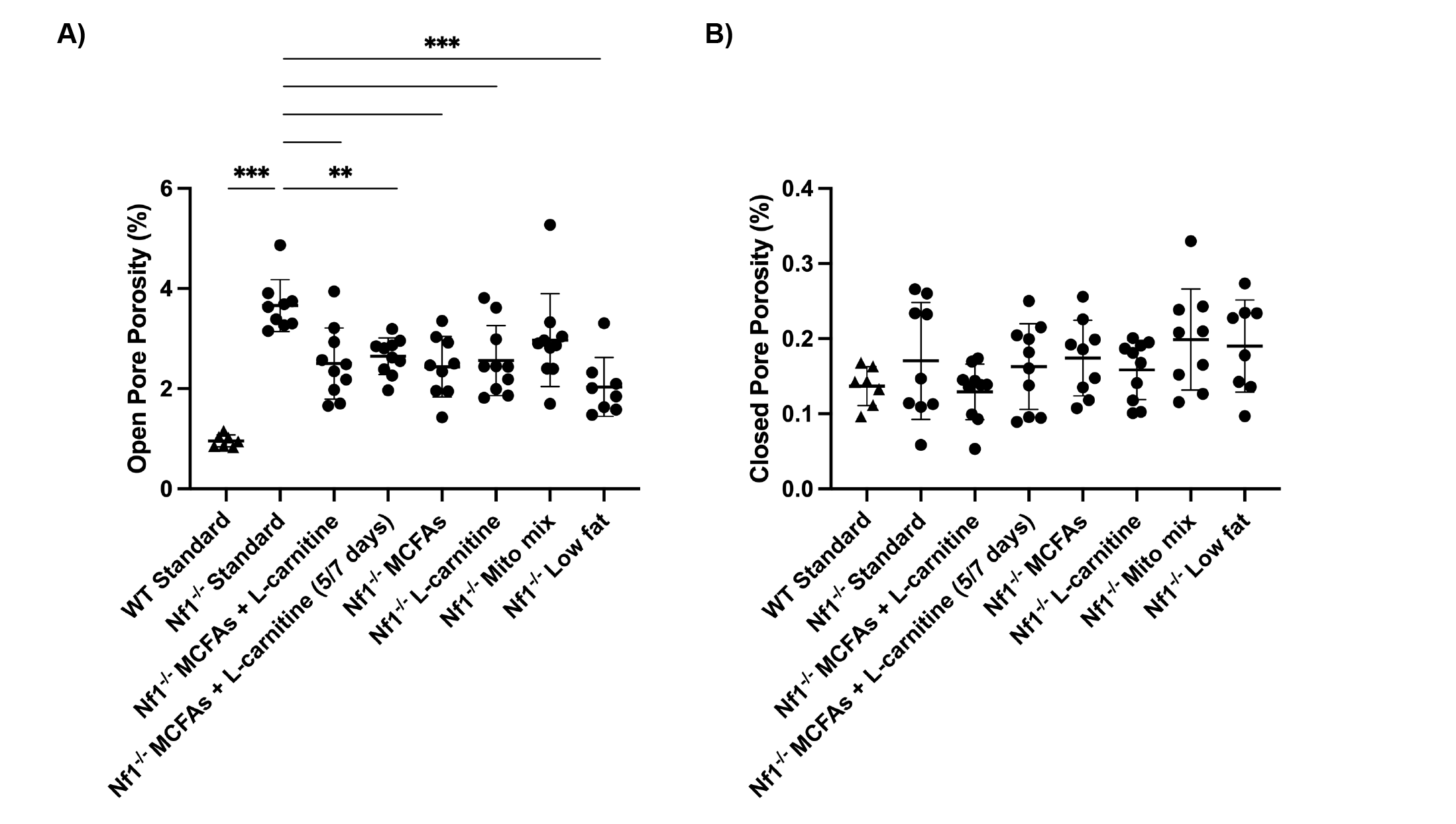

Supplement: S5 Fig — Outcome measures include (A) % open pore porosity (normalised by total volume), and (B) % closed pore porosity (normalized by total volume). Data were analysed using a one-way ANOVA with multiple comparison corrected by statistical hypothesis testing, * = p<0.05, ** = p<0.01, *** = p<0.001. (TIF) [file pone.0304778.s005.tif]

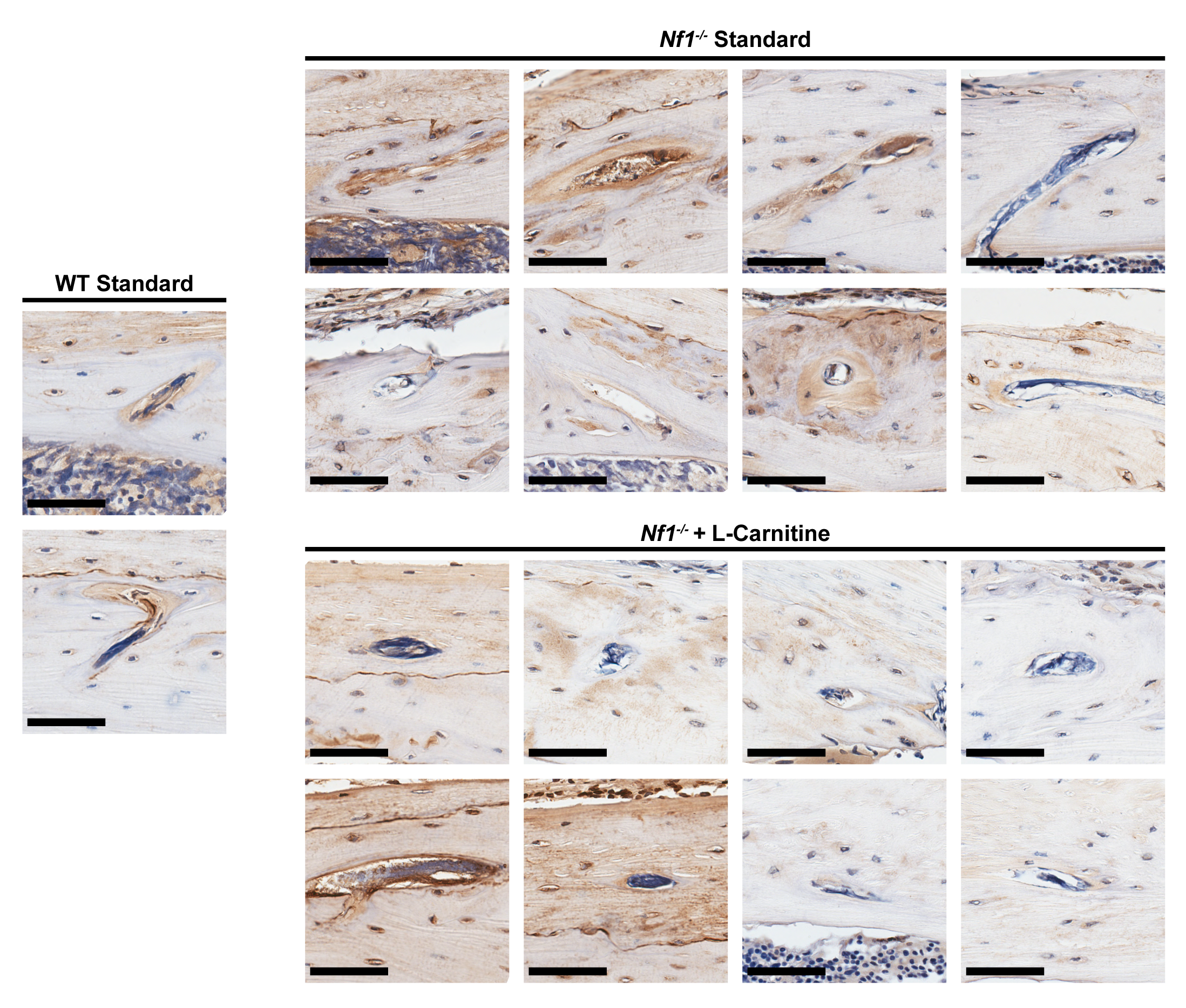

Supplement: S6 Fig — Descriptive histology illustrates the high variability of MGP staining both in the bone matrix and associated with large pores in the cortical bone. Scale bar represents 50 μm. (TIF) [file pone.0304778.s006.tif]
